# Development and external validation of a machine learning model for predicting gangrenous cholecystitis

**DOI:** 10.3389/fmed.2026.1930347

**Published:** 2026-09-14

**Authors:** Xianyue Ren, Liang Wang, Yue Zhu, Po Tang, Shan Cong

**Affiliations:** Department of General Surgery, The First Affiliated Hospital of Jinzhou Medical University, Jinzhou, Liaoning, China

**Keywords:** ANN model, cholecystitis, diagnostic predictive model, gangrenous cholecystitis, machine learning

## Abstract

**Background:**

Gangrenous cholecystitis (GC) is a severe surgical emergency with high postoperative morbidity and mortality, making early and accurate diagnosis essential for timely intervention. Conventional statistical models are limited in capturing nonlinear interactions among clinical variables and often lack interpretability, hindering individualized risk stratification. Machine learning enables accurate and interpretable risk prediction.

**Methods:**

The retrospective clinical study included a total of 492 patients with 39 routine serological tests and imaging tests collected. Seven models including LR, DT, RF, XGBoost, LightGBM, SVM and ANN were constructed. Model performance was evaluated using area under the curve (AUC), accuracy, sensitivity, specificity, and other metrics in train data, internal validation data and an independent external test data. An interpretable web-based risk calculator was dveloped based on the optimal model, with decile stratification employed to verify the reliability of predicted probabilities. SHapley Additive exPlanations (SHAP) were applied for feature interpretability.

**Results:**

The ANN model demonstrated the best performance, with an AUC of 0.932 (95% CI 0.900–0.961) in train data, 0.921 (95% CI 0.862–0.966) in internal validation data, and 0.867 (95% CI 0.790–0.929) in external validation. Five core predictors: NLR, WBC, FIB, NEU. and GBWT were identified as the most influential predictors. SHAP analysis provided interpretable insights into their contributions to infection risk. ANN model has been translated into a convenient tool to facilitate its utility in clinical settings.

**Conclusion:**

This study developed and externally validated an ANN-based prediction model for the early diagnosis of GC, achieving robust predictive performance and generalizability. The model holds promise for providing an early, personalized assessment of whether patients with cholecystitis will develop gangrene. Future prospective studies are warranted to further validate its efficacy.

## Introduction

Gangrenous cholecystitis (GC) is a severe form of acute cholecystitis (AC) and is characterized by gallbladder inflammation with eventual progressive necrosis of the wall and subsequent infection, initially localized and later becoming systemic (1–3). In patients with cholelithiasis, 1%–2% will develop cholecystitis per year, with a further estimated rate of GC reported in 2%–40% of cases of AC (4, 5), which has an incidence range of 2%–18% and a mortality rate between 15% and 50% (6–8). GC, as its symptoms may resemble those of uncomplicated AC, initially presents as a localized infection before progressing to systemic involvement. Timely surgical intervention, preferably within 72 h of symptom onset, correlates with less morbidity and abbreviated hospitalizations relative to postponed treatments (9–11). The severity of the condition directly impacts hospitalization duration, surgical rates, treatment costs, and prognosis. Therefore, early diagnosis of whether cholecystitis involves gangrene is crucial when patients seek medical attention. Current research indicates that only approximately 9% of GC patients receive an accurate diagnosis prior to surgery (12). Therefore, it's crucial to accurately assess whether patients with cholecystitis have developed gangrene at an early stage for selecting the appropriate treatment plan.

Since this disease can only be diagnosed through pathology evidence, it is hard to make a diagnosis for GC at an early stage. Numerous multidisciplinary studies have been conducted on how to diagnose GC at an early stage. A study have shown that the increase in-transient focal enhancement of the liver adjacent to the gallbladder during the arterial phase of dynamic CT was greater in GC than in non-gangrenous cholecystitis (NGC),and that arterial phase.

(ARAP) ≥ 1.46 was a predictor of GC (13). Marinova et al. thought age >65, male gender, diabetes mellitus, cardiovascular pathology, tachycardia > 90 bpm, white blood cells (WBC) > 14.10^9^/L, the thickness of gallbladder wall > 4 mm with pericholecystic fluid and C-Reactive Protein (CRP) > 150 mmg/L were the main predictors (14). Recently, Kler et al. found that Neutrophil-to-Lymphocyte Ratio (NLR) could be used to predict AC (15). However, most previous studies have employed traditional logistic regression models to predict diseases, resulting in a relatively large number of selected variables. Moreover, the majority of these variables require patients to undergo multiple tests, which significantly reduces the practical clinical application efficiency of the models.

With the rapid accumulation of multi-source clinical data—including laboratory test results, imaging findings, and electronic health records—machine learning (ML) has emerged as a powerful analytical tool for supporting clinical decision-making. In the field of clinical predictive modeling for various diseases, ML algorithms demonstrate unique advantages by enabling applications ranging from early screening and complication prediction to long-term prognosis assessment. These models are capable of effectively extracting latent nonlinear patterns from vast clinical datasets composed of demographic characteristics, biochemical markers, and imaging features. Furthermore, innovations in explainable AI technologies enable clinicians to trace the underlying rationale behind each predictive result, rather than merely obtaining a single risk score.

This study aimed to establish a cost-effective predictive model for GC based on ML approaches and develop a clinical decision support tool relying on the interpretability of the constructed model. It can provide clinicians with precise and evidence-based clinical decision assistance, and further optimize the diagnosis and treatment management of patients with GC.

## Materials and methods

### Study design and participants

This multicenter retrospective study was conducted in accordance with the Declaration of Helsinki (revised in 2013) and approved by the institutional review boards of all participating hospitals, with the requirement for informed consent waived due to the retrospective nature of the study. All patients were diagnosed with cholecystitis through ICD-9 code recognition and underwent cholecystectomy. This retrospective study was conducted at two Grade-A tertiary hospitals: the First Affiliated Hospital of Jinzhou Medical University and the Third Affiliated Hospital of Jinzhou Medical University, and approved by the Ethics Committee of two hospitals(2026LL-KY-316, KX2026026). Data from the First Affiliated Hospital of Jinzhou Medical University between September 1, 2024 and March 31, 2026 was randomly divided into a training set and a validation set (for internal validation) at a ratio of 7:3, while patients from the Third Affiliated Hospital of Jinzhou Medical University between September 1, 2025 and March 31, 2026 were served as an independent external test set. The study protocol is summarized in the flowchart (Figure 1).

**Figure 1:**
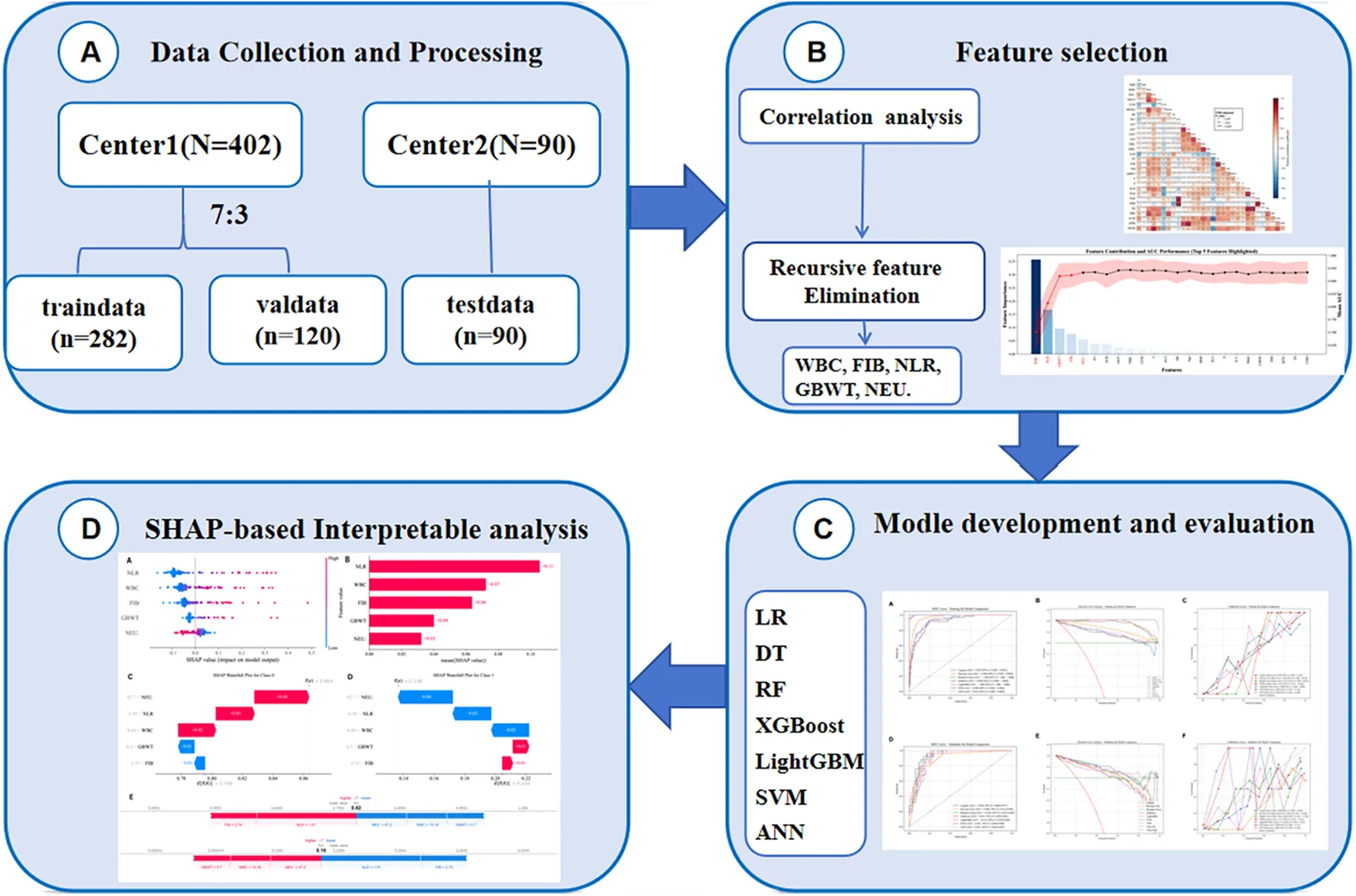
Flowchart of the overall study.

### Inclusion and exclusion criteria

The following are the inclusion and exclusion criteria for data collection:

Inclusion criteria:

(1) diagnosis of cholecystitis following the revised Tokyo guidelines 2018 (16), including positive Murphy's syndrome, peritoneal reaction or mass in right hypochondrium, signs of inflammation and data compatible with cholecystitis in B-mode ultrasonography; (2) performing cholecystectomy; (3) having complete and searchable clinical data; (4) not use antibiotics before admission.

Exclusion criteria:

(1) unavailable or incomplete medical records; (2) no surgical intervention; (3) malignancies;(4) individuals previously diagnosed with AC who had undergone percutaneous transhepatic gallbladder drainage (PTGBD) and were now admitted for elective laparoscopic cholecystectomy.

We confirm that both research centers strictly adhered to the continuous enrollment protocol. During the study period, all hospitalized patients who met the inclusion criteria were enrolled consecutively; no selective enrollment was performed based on disease severity, endpoint status, or other clinical characteristics. This enrollment strategy minimizes selection bias and ensures the clinical representativeness of the study cohort.

### Outcome and predictors

A comprehensive panel of candidate predictors was preselected based on clinical pathophysiological rationale and previously published evidence (2, 5, 17–20). All baseline data were collected within 24 h of admission, including clinical histories, physical examination findings, serological indices and ultrasonic parameters. The initial venous blood samples obtained on admission—prior to anti-infective treatment, fluid resuscitation and other interventions—were used for all laboratory measurements. Hematological data are analyzed using fully automated hematology analyzers, while liver function tests and coagulation biomarkers are measured using fully automated biochemical analyzers. All equipment undergoes regular internal quality control and national external quality assessment to ensure that the test results from both centers are stable and comparable.

The full variable set covered demographic characteristics (age,years; sex, BMI,kg/m^2^); admission vital signs: body temperature(T,℃) and heart rate(HR, beats/min); physical examination signs: tender point (TP),nausea and vomiting(N.V); comorbidities: presence of hypertension(HTN), coronary heart disease (CHD), diabetes mellitus(DM); complete blood count indices: white blood cell count(WBC,10^9^/L), neutrophil count(NEUT,10^9^/L), lymphocyte count(LYM,10^9^/L), monocyte count(MONO,10^9^/L), platelet count(PLT,10^9^/L), Neutrophil percentage(NEU., %), hemoglobin level(Hb,g/L), liver function and coagulation biomarkers: total bilirubin(TBIL,*μ*mol/L), direct bilirubin(DBIL,μmol/L), serum albumin(ALB,g/L), alkaline phosphatase(ALP,U/L),alanine aminotransferase (ALT,U/L), aspartate aminotransferase (AST,U/L), gamma-glutamyl transferase (GGT,U/L]), prothrombin time (PT,s), international normalized ratio (INR), fibrinogen levels(FIB,g/L) and color Doppler ultrasound measurements of gallbladder wall thickness(GBWT,cm).

The postoperative pathological results(PATH), as the primary outcome of this study, were issued by the Pathology Departments of the First and Third Affiliated Hospitals of Jinzhou Medical University; the pathological diagnosis of gastric cancer was confirmed as follows: full-thickness transmural necrosis of the gallbladder wall, accompanied by diffuse neutrophilic and mononuclear cell infiltration; supporting diagnostic features included gallbladder wall infarction and hemorrhage, as well as mucosal necrosis covered by inflammatory infiltrate and granulation tissue—these findings all meet the widely accepted pathological diagnostic criteria (21, 22). Both research centers adopted a unified primary endpoint definition for GC, namely the use of the ICD-10 diagnostic code K81.0, which ensured consistent case identification across both cohorts. For quality control purposes, each specimen was independently re-examined by two pathologists each with at least five years of pathological experience; all pathologists remained blinded to preoperative clinical data, laboratory test results, and group assignment information to avoid selection bias. In cases of disagreement regarding the initial diagnosis, a third pathologist conducted a re-evaluation, and the discrepancy was resolved through a consensus discussion within the pathology department. All reports were completed within seven days after cholecystectomy.

### Data preprocessing

After completing the preliminary data collection, this study included a patient dataset comprising data from 402 patients recruited from the First Affiliated Hospital of Jinzhou Medical University. First, prior to performing any data-based analytical operations, the dataset was randomly divided into a training cohort (70%) and an internal validation cohort (30%) (23). Next, all standardization, feature selection and hyperparameter optimization were restricted to the training data to prevent data leakage. The data constructed from the sample meeting the inclusion and exclusion criteria of this study contain no missing values, and the data are fully complete; therefore, multiple imputation was not employed in this study. Finally, continuous variables were standardized through Z-score normalization to improve model stability and reduce overfitting.

Given our plan to include 5–6 predictor variables and setting the event count (EPV) for each variable at 10, the sample size planning for the logistic regression model complies with the established “event count per variable” criterion (EPV=10) (24, 25). In addition, an independent external validation cohort was obtained from the Third Affiliated Hospital of Jinzhou Medical University (*n* = 90), which was used to assess the generalizability and robustness of the developed models. For all seven candidate models, their predictive factors were determined by employing a two-step fully quantitative feature screening process using the training set prior to model training; Step 1 FDR-adjusted Pearson correlation coefficient was performed to exclude severely collinear features; variables with |r|≥0.7 and adjusted *P* < 0.05 were filtered, and redundant items in each paired collinear set were removed. Step 2 Recursive Feature Elimination (RFE): Random forest-based five-fold cross-validation was adopted to rank all remaining features by predictive importance. We sequentially incorporated predictors from highest to lowest importance and recorded cross-validated AUC after each iteration. The feature set corresponding to the maximum cross-validated AUC was retained, as no further incremental improvement in discrimination was observed with additional variables, and the top optimal predictors were finalized for subsequent modeling. These predictors were used in seven machine learning models: logistic regression (LR), decision tree (DT), random forest (RF), LightGBM, XGBoost, support vector machine (SVM) and artificial neural network (ANN). Model performance was assessed using calibration curves, decision curve analysis (DCA), Receiver Operating Characteristic (ROC) curves and external validation.

### Machine learning algorithms

Seven machine learning algorithms were employed in this study: LR, DT, RF, SVM, ANN, XGBoost and LightGBM. Hyperparameter optimization was performed via grid search coupled with five-fold cross-validation. Six models achieved the best performance with parameters as followed in Table 1. Full hyperparameter tuning records for six models were shown in Supplementary Table S1. Meanwhile, 1,000-sample bootstrap resampling was implemented on the training set and internal validation set.

**Table 1:** Best hyperparameters of the 6 machine learning models for GC.

Modle	Best Hyperparameters
DT	'ccp*alpha': 0.0, 'max*depth': 5, 'max*features’: None, 'min*samples_split': 20
RF	n*estimators=450, max*features=2
XGBoost	'learning*rate': 0.1, 'max*depth': 3, 'n_estimators’: 100, ’subsample': 1.0
LightGBM	'colsample*bytree': 0.6, 'learning*rate': 0.2, 'n*estimators’: 100, 'num*leaves': 31, ’subsample': 0.6
SVM	'C': 0.1, 'degree': 2, 'gamma': ’scale', 'kernel': 'linear'
ANN	'activation': 'logistic', 'hidden*layer*sizes’: (100,)

### Model evaluation

Our study employed multiple evaluation metrics to assess model performance, including ROC curves, AUC, calibration curves, and DCA. ROC curves were plotted to evaluate the model's discriminatory ability, showing the trade-off between sensitivity and specificity at different threshold values. The AUC quantifies this trade-off, with a higher.

AUC indicating better overall performance in distinguishing between the positive and negative outcomes. Calibration curves were used to assess the agreement between predicted probabilities and actual outcomes, reflecting how well the model's predictions align with real-world results. DCA was performed to evaluate the clinical utility of the models by calculating the net benefit at different threshold probabilities, thus guiding the selection of optimal decision thresholds for clinical applications.

To evaluate the generalization capability of model, we conducted external validation using an independent dataset. This step ensures that the model's performance remains consistent across different populations and scenarios, further confirming its clinical applicability.

### SHAP interpretability analysis

To enhance the interpretability of the machine learning model,SHAP (Shapley Additive Explanations) values were calculated to quantify the contribution of each predictor to the model output. SHAP was based on Shapley values from cooperative game theory and estimated the contribution of each feature to the final prediction by considering different feature combinations. For each prediction, SHAP values indicated the direction and magnitude of a feature's effect on the outcome relative to the baseline prediction. In this study, SHAP values were calculated using the Kernel SHAP algorithm for the ANN model. The entire training cohort was adopted as the background dataset to derive the baseline prediction expectation, and SHAP values were computed for the validation cohort on the probability scale of GC. SHAP summary plots and dependence plots were used to visualize the overall importance and effect direction of each feature.

### Statistical analysis

Statistical analyses were conducted using R (v4.5.2) and Python (v3.10). Categorical variables were compared using the chi-square test. All continuous variables passed Shapiro–Wilk normality testing and showed non-normal distributions, thus all continuous data were presented as median (interquartile range, IQR). Intergroup comparisons between GC and NGC groups were performed with the Mann–Whitney U test. Two-sided *p*-values < 0.05 were considered statistically significant.

## Result

### Basic characteristics of the patients

The baseline characteristics of participants from the training cohort of the First Affiliated Hospital of Jinzhou Medical University (Table 2) and the external test cohort of the Third Affiliated Hospital of Jinzhou Medical University (Table 3) were summarized. None of the continuous variables followed a normal distribution. Significant differences were observed between the two cohorts in several variables. In both datasets, significant differences were observed in WBC, NEU., NEUT, LYM, MONO, DBIL, PT, INR, FIB, GBWT, HR, NLR, PLR, SII, SIRI, ALBI and NPAR(*p* < 0.05), among which, WBC, NEU., NEUT, LYM, MONO, PT, INR, NLR, SII, SIRI and NPAR in GC were strongly higher than NGC group(*p* < 0.001). At the training cohort, significant differences were also observed among patients in terms of Age, T and ALB (*p* < 0.001),however, there are no significant differences at the Third Affiliated Hospital of Jinzhou Medical University cohort. Morever, the baseline characteristics of the internal validation cohort were presented in Supplementary Table S2. Supplementary Table S3 displayed the intergroup comparison of baseline characteristics between the training and validation cohorts, which verified the balanced distribution of all baseline features following stratified random sampling. The comparison of baseline characteristics between two centers were calculated to quantify inter-centre distribution divergence in Supplementary Table S4.

**Table 2:** Baseline characteristics of patients in the training Set.

Variables	Overall(*N*=282)	NGC(*N*=222,78.72%)	GC(*N*=60,21.28%)	p
Gender[n(%)]				0.055
Female	160 (56.74)	133 (59.91)	27 (45.00)	
Male	122 (43.26)	89 (40.09)	33 (55.00)	
N.V [n(%)]				<0.001
No	171 (60.64)	151 (68.02)	20 (33.33)	
Yes	111 (39.36)	71 (31.98)	40 (66.67)	
DM [n(%)]				0.572
No	248 (87.94)	197 (88.74)	51 (85.00)	
Yes	34 (12.06)	25 (11.26)	9 (15.00)	
HTN [n(%)]				0.633
No	201 (71.28)	160 (72.07)	41 (68.33)	
Yes	80 (28.37)	61 (27.48)	19 (31.67)	
CHD [n(%)]				0.345
No	266 (94.33)	211 (95.05)	55 (91.67)	
Yes	16 (5.67)	11 (4.95)	5 (8.33)	
CBDS [n(%)]				0.557
No	230 (81.56)	179 (80.63)	51 (85.00)	
Yes	52 (18.44)	43 (19.37)	9 (15.00)	
TP [n(%)]				0.010
No	34 (12.06)	33 (14.86)	1 (1.67)	
Yes	248 (87.94)	189 (85.14)	59 (98.33)	
Age (years)	62.00 [49.25, 71.00]	60.50 [47.00, 69.00]	71.00 [61.00, 76.00]	<0.001
BMI(kg/m2)	24.80 [22.50, 26.38]	24.65 [22.30, 26.60]	24.90 [23.15, 26.12]	0.520
WBC(109/L)	7.39 [5.48, 11.09]	6.63 [5.21, 8.84]	12.43 [10.71, 16.35]	<0.001
NEU.(%)	73.30 [61.92, 86.17]	69.00 [59.10, 80.85]	87.85 [81.90, 91.48]	<0.001
NEUT (109/L)	5.30 [3.48, 9.27]	4.56 [3.32, 6.65]	10.92 [8.94, 14.41]	<0.001
LYM (109/L)	1.31 [0.88, 1.81]	1.41 [1.06, 1.90]	0.81 [0.68, 1.31]	<0.001
MONO (109/L)	0.36 [0.26, 0.56]	0.33 [0.24, 0.44]	0.61 [0.39, 0.83]	<0.001
Hb (g/L)	137.00 [126.00, 149.00]	136.50 [125.00, 147.75]	139.50 [127.75, 153.00]	0.218
PLT(109/L)	227.50 [188.00, 270.00]	229.50 [194.00, 267.75]	219.50 [174.00, 273.00]	0.206
ALT(U/L)	26.00 [16.00, 78.25]	27.00 [16.00, 80.75]	24.00 [16.00, 51.75]	0.494
AST (U/L)	25.50 [20.00, 50.00]	25.00 [20.00, 53.00]	26.00 [19.75, 43.25]	0.716
GGT (U/L)	44.88 [23.11, 190.75]	41.00 [21.89, 218.71]	52.66 [27.11, 134.34]	0.431
ALP (U/L)	102.00 [79.00, 142.00]	102.50 [76.25, 147.00]	99.50 [81.75, 132.50]	0.873
TBIL (μmol/L)	17.23 [12.17, 28.84]	16.82 [12.03, 26.80]	20.70 [12.78, 35.92]	0.139
DBIL (μmol/L)	5.25 [3.54, 11.50]	4.98 [3.50, 9.73]	7.00 [4.10, 16.99]	0.041
ALB (g/L)	42.95 [39.58, 45.30]	43.60 [40.60, 45.33]	40.09 [36.64, 43.97]	<0.001
PT (s)	11.90 [11.30, 12.60]	11.80 [11.20, 12.40]	12.50 [11.88, 13.77]	<0.001
INR	1.04 [0.98, 1.11]	1.03 [0.97, 1.08]	1.10 [1.04, 1.24]	<0.001
FIB (g/L)	3.39 [2.68, 4.96]	3.13 [2.55, 3.98]	5.96 [4.25, 7.50]	<0.001
GBWT (cm)	0.03 [0.03, 0.05]	0.03 [0.03, 0.03]	0.06 [0.04, 0.08]	<0.001
T (℃)	36.50 [36.30, 36.50]	36.50 [36.30, 36.50]	36.50 [36.30, 36.90]	<0.001
HR(beats/min)	80.00 [75.00, 89.00]	80.00 [74.00, 87.50]	84.00 [78.00, 98.50]	0.005
NLR	4.32 [2.06, 10.26]	3.06 [1.87, 6.06]	14.38 [7.46, 20.14]	<0.001
PLR	175.50 [126.01, 262.00]	164.93 [119.80, 226.81]	259.69 [173.43, 364.81]	<0.001
PAR	5.26 [4.48, 6.47]	5.26 [4.50, 6.34]	5.21 [4.27, 6.71]	0.994
PHR	1.61 [1.35, 1.97]	1.63 [1.40, 1.96]	1.53 [1.18, 1.99]	0.100
SII	900.44 [461.09, 2122.96]	715.71 [420.67, 1477.48]	2713.51 [1599.27, 4572.42]	<0.001
SIRI	1.23 [0.65, 4.98]	0.99 [0.56, 2.35]	8.19 [3.56, 15.93]	<0.001
ALBI	−2.58 [−2.85, −2.15]	−2.64 [−2.86, −2.32]	−2.15 [−2.61, −1.75]	<0.001
APRI	0.29 [0.21, 0.60]	0.28 [0.21, 0.60]	0.32 [0.23, 0.54]	0.718
NPAR	1.72 [1.42, 2.04]	1.61 [1.38, 1.90]	2.09 [1.93, 2.39]	<0.001

Categorical variables are presented as *n* (%) and compared using Chi-square test or Fisher exact test. Continuous variables are presented as median [interquartile range] and compared using Mann–Whitney U test. *P*< 0.05, which means they are statistically significant.

**Table 3:** Baseline characteristics of patients in the third affiliated hospital of jinzhou medical university cohort.

Variables	Overall(*N*=90)	NGC(*N*=66,73.33%)	GC(*N*=24,26.67%)	p
Gender[n(%)]				0.665
Female	39 (43.33)	30 (45.45)	9 ( 37.50)	
Male	51 (56.67)	36 (54.55)	15 ( 62.50)	
N.V [n(%)]				0.886
No	48 (53.33)	36 (54.55)	12 ( 50.00)	
Yes	42 (46.67)	30 (45.45)	12 ( 50.00)	
DM [n(%)]				0.455
No	67 (74.44)	51 (77.27)	16 ( 66.67)	
Yes	23 (25.56)	15 (22.73)	8 ( 33.33)	
HTN [n(%)]				0.886
No	48 (53.33)	36 (54.55)	12 ( 50.00)	
Yes	42 (46.67)	30 (45.45)	12 ( 50.00)	
CHD [n(%)]				0.382
No	81 (90.00)	61 (92.42)	20 ( 83.33)	
Yes	9 (10.00)	5 ( 7.58)	4 ( 16.67)	
CBDS [n(%)]				1.000
No	67 (74.44)	49 (74.24)	18 ( 75.00)	
Yes	23 (25.56)	17 (25.76)	6 ( 25.00)	
TP [n(%)]				0.100
No	10 (11.11)	10 (15.15)	0 ( 0.00)	
Yes	80 (88.89)	56 (84.85)	24 (100.00)	
Age (years)	64.50 [55.00, 71.00]	64.50 [51.25, 71.00]	65.50 [59.00, 73.00]	0.320
BMI(kg/m2)	24.78 (3.97)	24.49 (3.56)	25.58 (4.92)	0.251
WBC(109/L)	11.14 [7.31, 16.58]	9.16 [6.74, 13.58]	17.66 [12.11, 18.98]	<0.001
NEU.(%)	83.05 [67.50, 90.65]	75.65 [64.40, 87.40]	90.95 [86.83, 92.80]	<0.001
NEUT (109/L)	9.21 [4.93, 14.70]	7.05 [4.17, 11.67]	16.50 [10.11, 17.44]	<0.001
LYM (109/L)	1.14 [0.68, 1.63]	1.38 [0.94, 1.76]	0.64 [0.50, 1.03]	<0.001
MONO (109/L)	0.50 [0.36, 0.71]	0.43 [0.30, 0.64]	0.70 [0.56, 0.84]	<0.001
Hb (g/L)	141.50 [131.00, 150.75]	140.50 [129.25, 150.75]	144.00 [132.50, 148.00]	0.648
PLT(109/L)	234.00 [179.50, 306.00]	239.00 [194.50, 305.25]	222.00 [158.50, 307.00]	0.438
ALT(U/L)	33.50 [18.00, 142.50]	23.00 [17.00, 80.25]	73.50 [30.50, 326.75]	0.003
AST (U/L)	28.00 [18.25, 75.00]	22.50 [17.00, 60.75]	47.00 [30.25, 213.75]	0.001
GGT (U/L)	67.09 [29.17, 291.66]	52.00 [23.00, 223.75]	158.84 [57.83, 542.87]	0.011
ALP (U/L)	110.50 [85.25, 192.25]	96.00 [82.75, 152.50]	132.00 [94.00, 205.75]	0.239
TBIL (μmol/L)	22.75 [13.02, 38.66]	17.70 [12.50, 34.59]	33.96 [19.75, 79.67]	0.004
DBIL (μmol/L)	6.54 [3.82, 17.68]	4.60 [3.64, 13.50]	13.57 [7.12, 64.30]	0.002
ALB (g/L)	42.00 [37.12, 45.12]	42.64 [37.60, 45.61]	39.62 [36.08, 44.07]	0.079
PT (s)	12.20 [11.53, 12.90]	11.80 [11.43, 12.57]	12.80 [12.17, 13.95]	<0.001
INR	1.06 [1.00, 1.13]	1.03 [0.99, 1.10]	1.12 [1.06, 1.22]	<0.001
FIB (g/L)	4.17 [2.92, 5.88]	3.52 [2.79, 5.00]	5.86 [4.35, 7.34]	0.002
GBWT (cm)	0.50 [0.30, 0.70]	0.40 [0.30, 0.60]	0.60 [0.50, 0.80]	0.002
T (℃)	36.50 [36.40, 36.60]	36.50 [36.40, 36.60]	36.50 [36.40, 36.62]	0.783
HR(beats/min)	80.00 [75.00, 91.50]	79.00 [74.00, 89.75]	83.50 [77.50, 100.75]	0.048
NLR	7.44 [2.83, 19.29]	4.10 [2.35, 13.18]	21.41 [15.46, 30.27]	<0.001
PLR	211.34 [135.78, 316.05]	175.94 [120.47, 261.60]	288.23 [217.66, 402.30]	0.004
PAR	5.54 [4.42, 7.02]	5.54 [4.65, 7.02]	5.53 [4.22, 7.05]	0.781
PHR	1.61 [1.31, 2.23]	1.66 [1.35, 2.23]	1.48 [1.23, 2.18]	0.422
SII	2017.17 [626.27, 3959.76]	894.02 [552.40, 2943.52]	3850.23 [2427.04, 6762.83]	<0.001
SIRI	4.09 [1.23, 11.49]	2.46 [0.90, 5.72]	14.54 [8.09, 23.87]	<0.001
ALBI	−2.42 [−2.79, −1.83]	−2.50 [−2.82, −2.13]	−1.81 [−2.41, −1.53]	0.006
APRI	0.30 [0.20, 0.95]	0.25 [0.17, 0.74]	0.60 [0.28, 2.23]	0.005
NPAR	1.94 (0.50)	1.81 (0.48)	2.28 (0.39)	<0.001

Categorical variables are presented as *n* (%) and compared using Chi-square test or Fisher exact test. Continuous variables are presented as median [interquartile range] and compared using Mann–Whitney U test. P < 0.05, which means they are statistically significant.

### Feature selection

A two-step hybrid variable screening was conducted to optimize candidate predictors in the training set. First, Pearson correlation with FDR correction removed highly collinear variables (|r|≥0.7, FDR-adjusted *P* < 0.05) based on correlation heatmap (Figure 2A) and the redundant covariates, which were difficult to obtain clinically and relatively costly in terms of clinical application among the collinear variables, were excluded (5, 7, 17), thereby generating a preprocessed dataset. Second, random forest-based RFE sorted features by predictive importance, with 5-fold cross-validation calculating mean AUC at each incremental feature addition. As shown in Figure 2B, the optimal variable set was confirmed at the maximum cross-validated AUC, and five core predictors were retained for subsequent machine learning modeling. The five core predictors were WBC, NLR, GBWT, NEU. and FIB.

**Figure 2:**
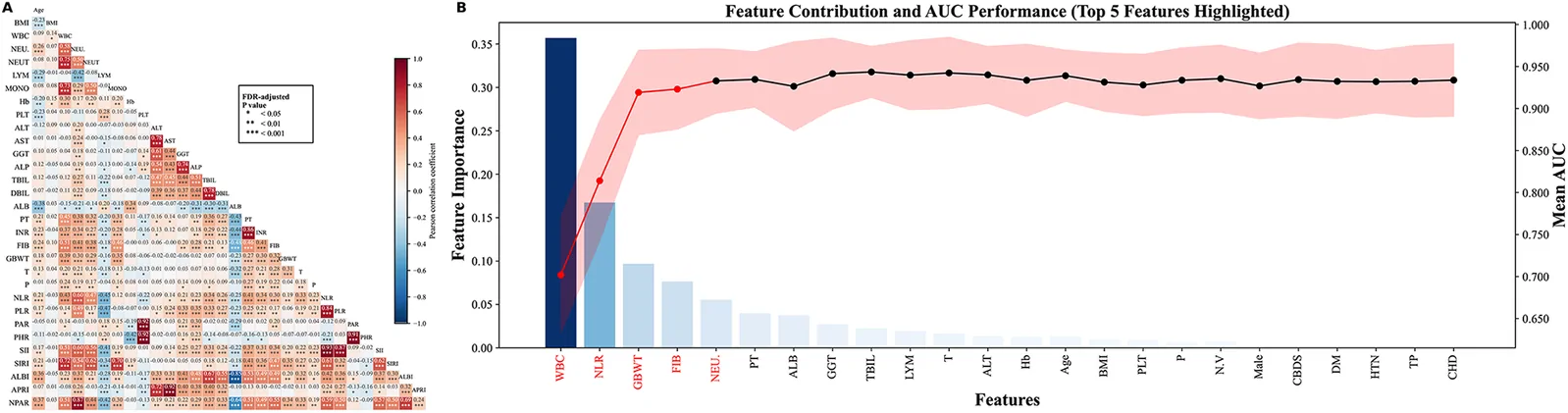
Feature selection using recursive feature elimination(RFE). **(A)** Correlation heatmap of all included continuous clinical covariates, red for positive correlation and blue for negative correlation; **(B)** The bar chart represents the sorted variables based on their importance to the classification task. The line chart delineated cumulative area under the receiver operating characteristic curves (AUCs) (right axis) on the inclusion of variables one by each iteration. Five predictors (coloured in red) were finally chosen for model development.

### Multimodel integrated analysis for classification

In this binary classification multi-model comparison study, we developed seven machine learning algorithms (including LR, DT, RF, XGBoost, LightGBM, SVM, and ANN) and evaluated these models using both the training set and the internal validation set. The study employed a comprehensive set of multidimensional metrics—namely, AUC, sensitivity, specificity, PPV, NPV, accuracy, balanced accuracy, F1 score, Brier score, calibration intercept, calibration slope, and Observed-to-Expected ratio (O/E ratio)—to comprehensively assess each model's discriminative ability. By combining the internal validation set (Table 4) with the calibration curve (Figure 3), we comprehensively evaluate each model's discriminative ability, balanced classification performance and calibration level.

**Table 4:** Performance metrics of machine learning models on the validation set.

Model	LR	DT	RF	XGBoost	LightGBM	SVM	ANN
AUC	0.936 [0.889, 0.977]	0.890 [0.815, 0.947]	0.924 [0.877, 0.964]	0.912 [0.861, 0.958]	0.914 [0.858, 0.956]	0.921 [0.866, 0.966]	0.921 [0.862, 0.966]
Sensitivity	0.760 [0.583, 0.913]	0.800 [0.630, 0.952]	0.720 [0.542, 0.895]	0.720 [0.536, 0.882]	0.560 [0.360, 0.741]	0.640 [0.440, 0.811]	0.720 [0.500, 0.895]
Specificity	0.937 [0.889, 0.980]	0.863 [0.798, 0.925]	0.884 [0.814, 0.945]	0.853 [0.781, 0.924]	0.884 [0.813, 0.946]	0.926 [0.866, 0.978]	0.916 [0.857, 0.968]
PPV	0.760 [0.579, 0.926]	0.606 [0.436, 0.765]	0.621 [0.433, 0.792]	0.562 [0.400, 0.742]	0.560 [0.348, 0.762]	0.696 [0.515, 0.895]	0.692 [0.500, 0.870]
NPV	0.937 [0.882, 0.979]	0.943 [0.888, 0.988]	0.923 [0.863, 0.977]	0.920 [0.864, 0.967]	0.884 [0.816, 0.940]	0.907 [0.845, 0.959]	0.926 [0.867, 0.978]
Accuracy	0.900 [0.842, 0.950]	0.850 [0.791, 0.908]	0.850 [0.783, 0.908]	0.825 [0.758, 0.892]	0.817 [0.742, 0.883]	0.867 [0.800, 0.925]	0.875 [0.808, 0.933]
Balanced Accuracy	0.848 [0.756, 0.929]	0.832 [0.742, 0.911]	0.802 [0.707, 0.896]	0.786 [0.695, 0.876]	0.722 [0.620, 0.818]	0.783 [0.681, 0.881]	0.818 [0.711, 0.910]
F1 Score	0.760 [0.611, 0.880]	0.690 [0.538, 0.811]	0.667 [0.514, 0.800]	0.632 [0.481, 0.769]	0.560 [0.381, 0.714]	0.667 [0.500, 0.818]	0.706 [0.529, 0.836]
Brier Score	0.088 [0.052, 0.126]	0.117 [0.071, 0.165]	0.094 [0.062, 0.127]	0.113 [0.074, 0.155]	0.142 [0.092, 0.200]	0.097 [0.066, 0.132]	0.092 [0.060, 0.128]
Calibration Intercept	−0.610 [−1.292, 0.185]	−0.502 [−1.119, 0.160]	−0.425 [−1.132, 0.220]	−0.432 [−1.064, 0.368]	−0.256 [−0.933, 0.449]	−0.405 [−1.069, 0.573]	−0.251 [−0.946, 0.433]
Calibration Slope	0.878 [0.624, 1.543]	0.218 [0.138, 0.455]	0.867 [0.650, 1.358]	0.677 [0.514, 0.968]	0.284 [0.218, 0.383]	1.094 [0.777, 1.850]	1.004 [0.728, 1.578]
O/E Ratio	0.820 [0.623, 1.019]	0.853 [0.626, 1.102]	0.860 [0.638, 1.080]	0.893 [0.652, 1.162]	1.002 [0.706, 1.351]	0.812 [0.623, 1.030]	0.902 [0.660, 1.146]

**Figure 3:**
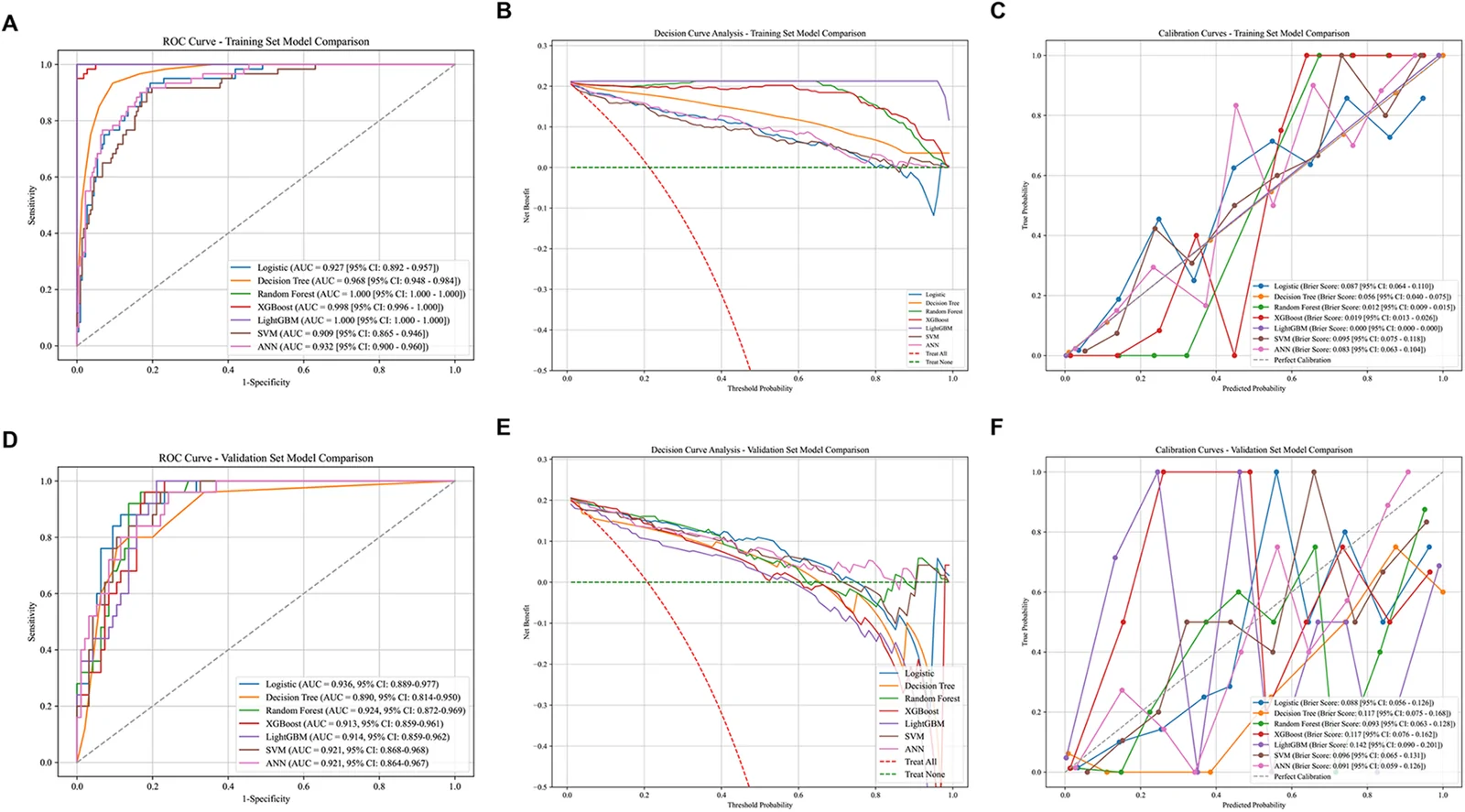
The performance and comparison of multiple predictive models. **(A)** ROC Curves for the training Set; **(B)** DCA curves for the training Set; **(C)** Calibration Curve for the training Set; **(D)** ROC Curves for the validation Set; **(E)** DCA curves for the validation Set; **(F)** Calibration Curve for the validation Set.

Both RF and LightGBM achieved theoretical optimal performance on the training set, with their AUC, sensitivity, specificity, and F1 score all reaching 1.000. However, when applied to the validation set, their performance declined significantly: the AUC of RF on the validation set dropped to 0.924, while its balanced accuracy was only 0.802. For LightGBM, the AUC on the validation set fell to 0.914, and its balanced accuracy plummeted to 0.722. LightGBM exhibited the poorest calibration among all models, with a high Brier score of 0.142 on the validation set and a calibration slope of merely 0.284, as illustrated by the calibration curve in Figure 3, which intuitively demonstrates its prediction bias; DT achieved the lowest AUC on the validation set at 0.890, with a calibration slope of 0.218, showing a severe deviation from the ideal level; XGBoost's balanced accuracy on the validation set was only 0.786, indicating suboptimal overall classification performance; SVM demonstrated stable specificity, but its sensitivity on the training set was only 0.517 and on the validation set was 0.640, reflecting insufficient balanced predictive capability.

The ANN model exhibits the smallest performance degradation when transitioning from the training set to the validation set, with comprehensive metrics remaining stable and balanced: for the training set, AUC=0.932, balanced accuracy=0.810, and F1 score=0.713; for the validation set, AUC=0.921, balanced accuracy=0.818, and F1 score=0.706. In terms of calibration performance, the calibration slope of the ANN model on the validation set is 1.004—nearly identical to the ideal value of 1—with a Brier score of 0.092; as shown in Figure 3, the ANN calibration curve closely aligns with the ideal diagonal line. The O/E ratio for the training set is 1.041, and the O/E ratio for the validation set is 0.902; both values fluctuate slightly around 1, indicating no systematic overestimation or underestimation of risk probabilities and demonstrating the robustness of the model's calibration performance.

The ANN model struck a balance among its discriminative performance, cross-subset generalization stability, and calibration capability, achieving optimal overall performance; therefore, this study ultimately selected the ANN as the predictive model for subsequent analyses. Finally, the detailed parameters of the ANN model are provided in Supplementary Table S5.

### External test Set validation

External validation was performed on the finalized ANN model using an independent external cohort of 90 patients. Table 5 summarized all quantitative discrimination and calibration metrics with corresponding 95% confidence intervals (CIs), and the model yielded an AUC of 0.867 [0.790, 0.929]. Figure 4 integrates the ROC curve, calibration curve along with a 95% uncertainty interval and DCA to comprehensively assess the predictive performance of the model on real-world external samples. The confusion matrix for the external validation set was provided in Supplementary Figure S1 and the classification threshold was 0.5. For calibration evaluation (Figure 4A), the model had a Brier score of 0.146 [0.105, 0.193] and a calibration intercept of −0.995 [−1.772, −0.446]; The 95% confidence interval intuitively illustrates the range of fluctuation in the model's predicted risk. As shown in Figure 4C, quantitative DCA calculations revealed that the ANN model achieved greater net clinical benefit than the two reference management strategies (presuming no gangrene for all patients, diagnosing GC for all patients) within the threshold probability range of 0–0.58.

**Table 5:** Performance metrics of machine learning models on external test set.

Model	ANN
AUC	0.867 [0.790, 0.929]
Sensitivity	0.917 [0.800, 1.000]
Specificity	0.742 [0.629, 0.844]
PPV	0.564 [0.400, 0.711]
NPV	0.961 [0.902, 1.000]
Accuracy	0.789 [0.700, 0.867]
Balanced Accuracy	0.830 [0.747, 0.897]
F1 Score	0.698 [0.552, 0.812]
Brier Score	0.146 [0.105, 0.193]
Calibration Intercept	−0.995 [−1.772, −0.446]
Calibration Slope	1.053 [0.745, 1.604]
O/E Ratio	0.686 [0.486, 0.870]

**Figure 4:**
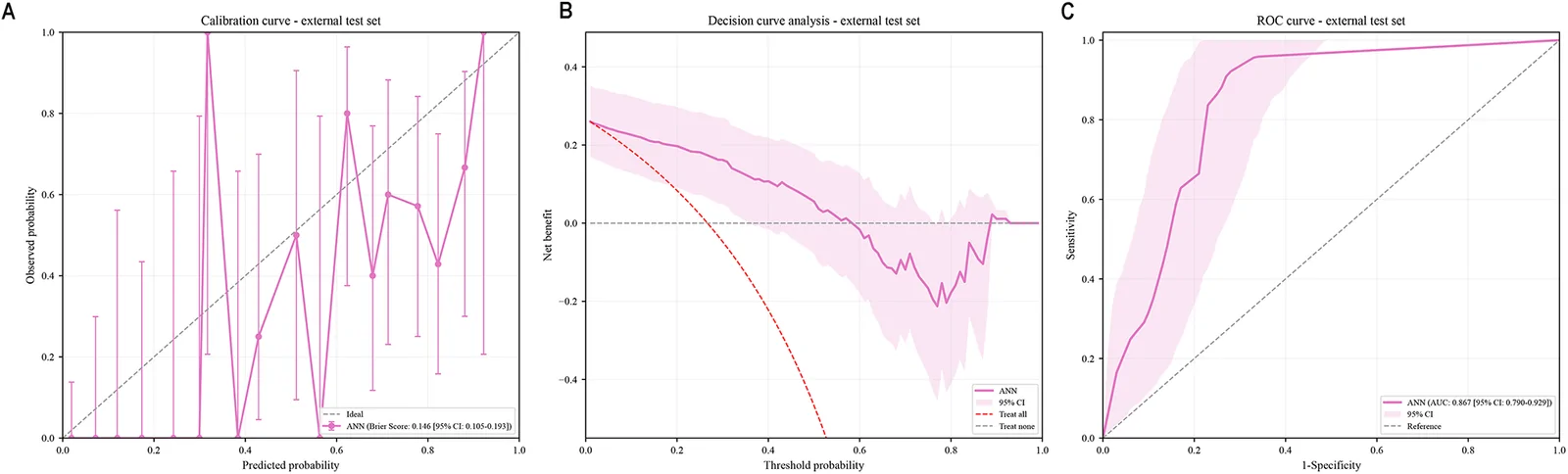
Evaluation of the ANN model performance. **(A)** Calibration curve: The *y*-axis represents the true probability and the *x*-axis represents the predicted probability. The model demonstrated a Brier score of 0.146 (95% CI: 0.105–0.193). **(B)** DCA curves for the external test set. **(C)** ROC curve: The area under the curve (AUC) is 0.867 (95% CI: 0.790–0.929), indicating strong predictive accuracy.

The ANN model demonstrated slightly reduced performance on unseen external patient samples compared to its internal validation set, yet generally maintained stable performance. However, due to the limited sample size of the external validation cohort, the risk estimates may lack stability, and the clinically meaningful DCA threshold range may exhibit variability. Therefore, caution should be exercised when interpreting its calibration reliability and clinical decision-making value. Future research should conduct large-scale, multicenter external validations to further confirm its generalizability as a clinical screening tool.

### SHAP interpretability analysis

The clinical utility and interpretability of the ANN model were further elucidated through SHAP visualizations as shown in Figure 5. Global analysis (Figures 5A,B) identified NLR as the most influential predictor, followed by WBC, FIB, GBWT, and NEU. Waterfall and force plots (Figures 5C–E) illustrated individual predictions, showing how each feature contributed to shifting the model's output from the baseline toward the final risk estimate. This interpretability ensures that the model is not merely a black box but a transparent clinical application that meets the study objective of supporting personalized treatment strategies for patients with cholecystitis.

**Figure 5:**
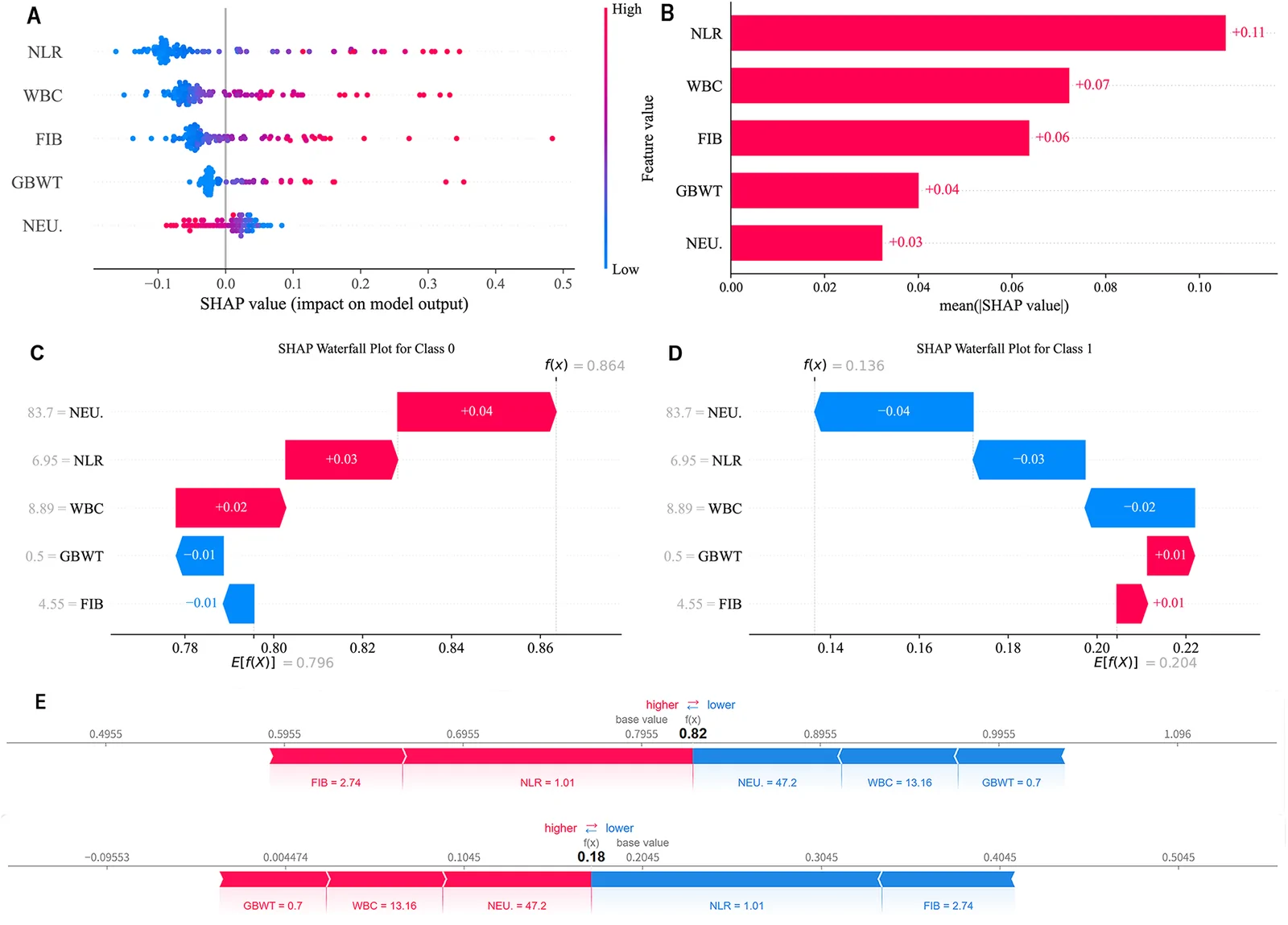
SHAP of the ANN model. **(A)** SHAP summary showing feature contributions, with red and blue dots representing higher and lower values, respectively;**(B)** SHAP importance bar chart ranked by the mean absolute SHAP value of each predictor; **(C,D)** SHAP waterfall plots for Class 0 and Class 1 subgroups to quantify individual cumulative feature contribution to prediction output (Class 0 = NGC, Class 1 = GC); **(E)** SHAP decision plots illustrating the overall predictive contribution trend of each variable.

### Webpage deployment tool

We have integrated a ANN model into a web application (Figure 6). When users provide the actual values of the five features required by the model, the application automatically predicts an individual's probability of developing GC and displays this risk value on a waterfall chart and a force plot, thereby enabling an intuitive visual representation. This web application can be accessed online at https://med.columbina.eu.org.

**Figure 6:**
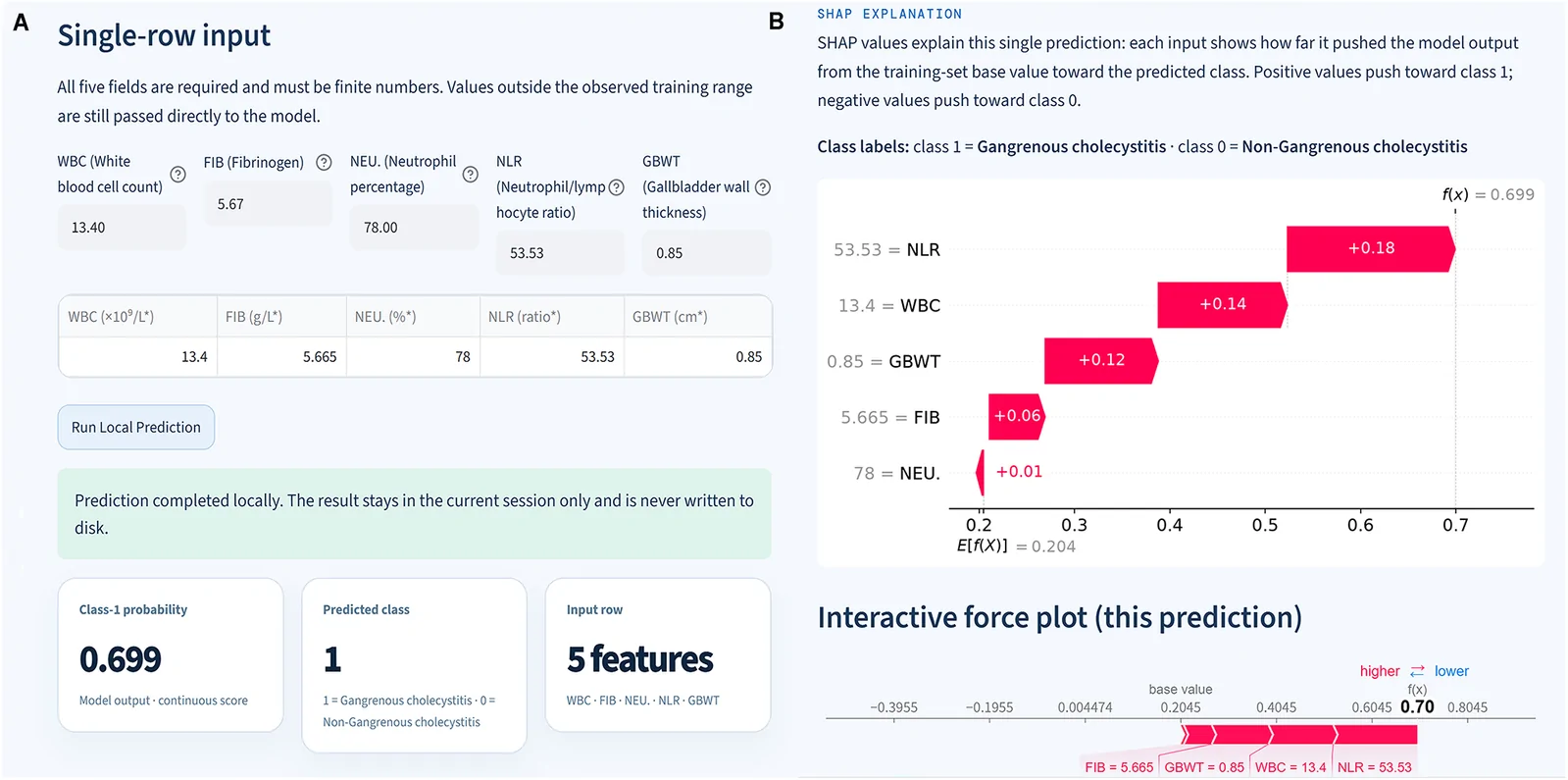
Web-based applications suitable for clinical practice. **(A)** Input five clinical variables to output the corresponding predictive probability; **(B)** SHAP waterfall plot and force plot for Class 1 of representative samples.

## Discussion

This study presents a novel predictive tool for early detection of GC. Our final model identified five key predictors, including WBC, NLR, GBWT, NEU. and FIB, and achieved achieved excellent overall performance on both the internal validation set and the external validation set, demonstrating strong discriminative power and excellent generalization capability.

Our study results indicate that GC is significantly correlated with NLR, WBC, and FIB, particularly with NLR, as demonstrated by the Shap analysis, aligns with the work of previous research (10, 19, 26). In previous studies, NLR was proposed as a biomarker for systemic inflammation and stress. Different acute inflammatory diseases like appendicitis, abscesses, septic shock are followed by increased NLR, also seen in critically ill trauma patients (27). The most severe and marked elevation of neutrophil counts and very deep decline in lymphocyte counts were observed in association with most severe critical illnesses such as septic shock, hemorrhagic shock, multiple trauma, and acute pancreatitis (28)，which explains the etiology of elevated NLP levels when a patient with cholecystitis develops gangrene. Meanwhile, Bouassida et al. reported NLR > 5 was a still independent factor for gangrene through univariate and multivariate analysis. Both NLR and FIB are pivotal variables determining the prediction direction: even if other variables have low values, the outcome will still fall into the high-probability zone as long as these two variables are elevated, and vice versa. Our results are consistent with those of Ma et al. (26). Although we employed a recursive elimination approach to screen variables and Ma et al. utilized Lasso regression analysis, WBC, NLR, GBWT, and FIB were screened into final model constructions and achieved favorable validation results. It confirms that these variables demonstrate significant predictive value for early detection of GC. Additionally, within the model, we found that an elevated NEU. level reduced the predicted probability, which is inconsistent with previous studies (19, 29). We hypothesize that fully established gangrenous cholecystitis triggers massive neutrophil chemotaxis and sequestration within necrotic gallbladder tissue, leading to peripheral circulating neutrophil depletion and reduced measurable NEU. in severe late-stage disease (30). As for WBC,which is routine testing indicators for infectious diseases. As key components of the immune defense system, WBC eliminate invasive pathogens via phagocytosis and antibody production. Bacterial infection typically induces elevated WBC levels (31). The ANN model is consistent with it.

With the application of machine learning in medicine, an increasing number of clinical prediction models based on machine learning have been developed. Several prediction models and risk scores for GC have been proposed. These studies generally demonstrated good discrimination but were predominantly single-center, rarely included external validation or formal assessment of calibration and clinical net benefit, and a relatively large number of clinical predictive indicators. Hu et al. developed a RF model comprising 10 variables with AUC of 0.875 (21). Ma et al. developed a XGBoost ML model comprising 7 variables with AUC of 0.944 (26). Chen et al. screened 3 predictive indexes: WBC, GBWT, the percentage of neutrophils using one-way ANOVA (19). Our model builds on this literature by using only preoperative, routinely collected 5 variables, which can be obtained by routine blood sampling and hepatobiliary ultrasound, validating performance in an external hospital cohort, and providing a comprehensive evaluation that includes discrimination, calibration plots, and decision-curve analysis. Meanwhile,it is the first time that the only five predictors were combined to establish a GC risk prediction ANN model. More importantly, we have integrated the ANN model into a web-based application. This tool offers auxiliary reference for the early identification of gangrenous change in cholecystitis patients at their initial presentation, enabling clinicians to deliver individualized therapeutic strategies accordingly.

Despite these strengths, the model still has several shortcomings. First, we constructed and externally validated an ANN preoperative prediction model for GC using five routine clinical indicators, which showed favorable discrimination and calibration in internal and single affiliated-hospital validation. However, external testing was only conducted at a geographically adjacent tertiary hospital affiliated with the same medical university. The two centres share overlapping regional patient populations, unified laboratory and ultrasound testing protocols, and relatively homogeneous study participants, which may partially inflate the observed AUC values and cannot confirm broad nationwide or international generalizability of the model. Large-scale prospective multicentre validation covering diverse regions and all tiers of medical institutions, alongside formal clinical impact studies measuring patient-level prognostic changes, are mandatory before this risk calculator can be widely recommended for routine emergency biliary surgery decision-making.

In conclusion, we successfully developed and externally validated an ANN model for the early diagnosis of GC, achieving high classification accuracy of 90.00% and AUC of 93.20% in the training cohort. The model provides an individualized and precise diagnostic approach for the early diagnosis of clinical GC.These findings support the application of machine learning in personalized clinical management.

## Conclusion

We developed and externally validated an interpretable ANN prediction model for the preoperative diagnosis of GC based on five routine laboratory and ultrasound indicators. The model achieved favorable discrimination and calibration performance across both the internal validation cohort and external test cohort. This web-based risk calculator enables individualized risk stratification and provides auxiliary evidence for clinicians in the early diagnosis of GC. Nevertheless, the external validation was only conducted at a single nearby tertiary hospital within the same city, and the retrospective study design limits its widespread clinical implementation. Large-scale prospective multicenter validation covering diverse medical settings, alongside clinical outcome impact studies, is mandatory to determine the optimal clinical decision threshold before this predictive tool can be routinely recommended for clinical practice.

## Data Availability

The original contributions presented in the study are included in the article/Supplementary Material, further inquiries can be directed to the corresponding author.
