# Supplementary material for: Development and external validation of a machine learning model for predicting gangrenous cholecystitis
Source: Front Med (Lausanne). 2026 Sep 14;13:1930347. doi: 10.3389/fmed.2026.1930347 (PMC13616794; doi:10.3389/fmed.2026.1930347)
Supplement: Supplementary file 1 [file Table1.docx]

Supplementary Table 1 Full hyperparameter tuning records for six comparative models

| **Model** | **Software package** | **Hyperparameter search space** | **Optimal hyperparameters** | **Tuning strategy** |
| --- | --- | --- | --- | --- |
| Decision Tree | scikit-learn | max_depth: [3, 5, 10, None]; min_samples_split: [5, 10, 20]; max_features: [sqrt, None]; ccp_alpha: [0.0, 0.01, 0.1] | ccp_alpha = 0.0; max_depth = 5; max_features = None; min_samples_split = 20 | 5-fold cross-validation grid search, optimized for ROC-AUC |
| Random Forest | scikit-learn | n_estimators: 50–450 (interval = 50); max_features: [2] | n_estimators = 450; max_features = 2 | 5-fold cross-validation grid search, optimized for ROC-AUC |
| XGBoost | XGBoost Python package | learning_rate: [0.01, 0.1, 0.2]; max_depth: [3, 5, 10]; n_estimators: [50, 100, 200]; subsample: [0.6, 0.8, 1.0] | learning_rate = 0.1; max_depth = 3; n_estimators = 100; subsample = 1.0 | 5-fold cross-validation grid search, optimized for ROC-AUC |
| LightGBM | LightGBM Python package | learning_rate: [0.1, 0.2]; num_leaves: [31, 50, 100]; n_estimators: [100]; subsample: [0.6, 0.8, 1.0]; colsample_bytree: [0.6, 0.8, 1.0] | colsample_bytree = 0.6; learning_rate = 0.2; n_estimators = 100; num_leaves = 31; subsample = 0.6 | 5-fold cross-validation grid search, optimized for ROC-AUC |
| SVM | scikit-learn | C: [0.1]; kernel: [linear, rbf]; gamma: [scale, auto, 0.01]; degree: [2, 3, 4] | C = 0.1; degree = 2; gamma = scale; kernel = linear | 5-fold cross-validation grid search, optimized for ROC-AUC |
| ANN | scikit-learn | hidden_layer_sizes: [(25,), (50,), (100,), (10, 10), (50, 50), (100, 100), (50, 50, 50)]; activation: [relu, tanh, logistic] | activation = logistic; hidden_layer_sizes = (100,) | 5-fold cross-validation grid search, optimized for ROC-AUC |

All model construction and statistical analyses were implemented in Python 3.10. Core dependency versions: scikit-learn 1.3.2, XGBoost 2.0.3, LightGBM 4.1.0, pandas 2.0.3, NumPy 1.24.4. A fixed random seed (random_state = 123) was applied to all algorithms to ensure full reproducibility. All hyperparameter tuning was conducted exclusively on the training set to eliminate data leakage.
