# Supplementary material for: Development and external validation of a machine learning model for predicting gangrenous cholecystitis
Source: Front Med (Lausanne). 2026 Sep 14;13:1930347. doi: 10.3389/fmed.2026.1930347 (PMC13616794; doi:10.3389/fmed.2026.1930347)
Supplement: Supplementary file 2 [file Table2.docx]

**Supplementary Table 2. Baseline Characteristics of Patients in the Internal Validation Set.**

| **Variables** | **Overall（N=120）** | **NGC（N=95,79.17%）** | **GC（N=25,20.83%）** | **p** |
| --- | --- | --- | --- | --- |
| Gender[n(%)] |  |  |  | <0.001 |
| Female | 67 (55.83) | 63 (66.32) | 4 (16.00) |  |
| Male | 53 (44.17) | 32 (33.68) | 21 (84.00) |  |
| N.V [n(%)] |  |  |  | 0.555 |
| No | 71 (59.17) | 58 (61.05) | 13 (52.00) |  |
| Yes | 49 (40.83) | 37 (38.95) | 12 (48.00) |  |
| DM [n(%)] |  |  |  | 0.399 |
| No | 96 (80.00) | 78 (82.11) | 18 (72.00) |  |
| Yes | 24 (20.00) | 17 (17.89) | 7 (28.00) |  |
| HTN [n(%)] |  |  |  | 0.088 |
| No | 86 (71.67) | 72 (75.79) | 14 (56.00) |  |
| Yes | 34 (28.33) | 23 (24.21) | 11 (44.00) |  |
| CHD [n(%)] |  |  |  | 0.392 |
| No | 111 (92.50) | 89 (93.68) | 22 (88.00) |  |
| Yes | 9 (7.50) | 6 (6.32) | 3 (12.00) |  |
| CBDS [n(%)] |  |  |  | 1.000 |
| No | 97 (80.83) | 77 (81.05) | 20 (80.00) |  |
| Yes | 23 (19.17) | 18 (18.95) | 5 (20.00) |  |
| TP [n(%)] |  |  |  | 0.155 |
| No | 23 (19.17) | 21 (22.11) | 2 (8.00) |  |
| Yes | 97 (80.83) | 74 (77.89) | 23 (92.00) |  |
| Age (years) | 64.50 [50.00, 71.00] | 62.00 [47.50, 70.00] | 66.00 [59.00, 78.00] | 0.010 |
| BMI(kg/m^2^) | 24.75 [22.00, 27.07] | 24.80 [21.95, 27.30] | 24.40 [23.40, 26.00] | 0.670 |
| WBC(10^9^/L) | 7.96 [5.74, 12.35] | 6.99 [5.31, 10.35] | 13.74 [9.87, 18.59] | <0.001 |
| NEU.(%) | 72.80 [66.75, 85.72] | 71.00 [63.70, 79.05] | 89.00 [83.40, 91.00] | <0.001 |
| NEUT (10^9^/L) | 5.94 [3.70, 10.29] | 5.04 [3.43, 7.36] | 12.28 [8.79, 16.54] | <0.001 |
| LYM (10^9^/L) | 1.31 [0.90, 1.77] | 1.41 [1.06, 1.86] | 0.99 [0.56, 1.40] | 0.002 |
| MONO (10^9^/L) | 0.38 [0.28, 0.59] | 0.35 [0.27, 0.46] | 0.71 [0.43, 0.99] | <0.001 |
| Hb (g/L) | 138.50 [130.00, 147.00] | 138.00 [128.00, 146.00] | 142.00 [137.00, 157.00] | 0.024 |
| PLT(10^9^/L) | 233.50 [181.75, 284.25] | 236.00 [192.00, 285.00] | 221.00 [179.00, 278.00] | 0.268 |
| ALT(U/L) | 24.00 [14.75, 57.00] | 25.00 [14.00, 75.00] | 22.00 [17.00, 32.00] | 0.311 |
| AST (U/L) | 23.00 [18.00, 37.25] | 24.00 [19.00, 39.50] | 22.00 [18.00, 30.00] | 0.189 |
| GGT (U/L) | 39.00 [19.03, 173.25] | 38.00 [18.64, 198.62] | 39.38 [25.80, 76.00] | 0.703 |
| ALP (U/L) | 97.00 [80.25, 143.25] | 98.00 [81.00, 166.00] | 96.00 [78.00, 120.00] | 0.453 |
| TBIL (μmol/L) | 15.80 [11.93, 23.30] | 15.00 [12.00, 21.55] | 22.60 [11.97, 33.78] | 0.165 |
| DBIL (μmol/L) | 4.66 [3.76, 8.80] | 4.40 [3.73, 6.95] | 7.60 [4.30, 10.83] | 0.069 |
| ALB (g/L) | 42.67 [39.08, 45.28] | 43.54 [40.60, 45.70] | 38.89 [36.04, 40.60] | <0.001 |
| PT (s) | 11.85 [11.20, 12.70] | 11.60 [11.20, 12.30] | 13.40 [12.60, 14.50] | <0.001 |
| INR | 1.04 [0.98, 1.12] | 1.02 [0.97, 1.08] | 1.18 [1.10, 1.27] | <0.001 |
| FIB (g/L) | 3.45 [2.72, 4.93] | 3.10 [2.65, 4.29] | 6.33 [4.07, 7.50] | <0.001 |
| GBWT (cm) | 0.03 [0.03, 0.05] | 0.03 [0.03, 0.04] | 0.06 [0.05, 0.07] | <0.001 |
| T (℃) | 36.50 [36.50, 36.60] | 36.50 [36.50, 36.55] | 36.50 [36.50, 36.60] | 0.204 |
| HR(beats/min) | 79.00 [74.00, 86.25] | 79.00 [74.00, 85.50] | 78.00 [75.00, 93.00] | 0.543 |
| NLR | 3.79 [2.53, 10.37] | 3.12 [2.25, 5.55] | 14.45 [7.80, 22.19] | <0.001 |
| PLR | 177.20 [139.57, 252.87] | 161.24 [136.69, 241.06] | 219.33 [175.17, 341.51] | 0.015 |
| PAR | 5.47 [4.62, 6.74] | 5.49 [4.55, 6.55] | 5.40 [4.71, 7.01] | 0.737 |
| PHR | 1.69 [1.35, 2.06] | 1.71 [1.38, 2.08] | 1.46 [1.12, 1.92] | 0.066 |
| SII | 1008.31 [556.11, 2214.86] | 811.04 [522.37, 1373.07] | 3070.17 [1412.89, 4382.16] | <0.001 |
| SIRI | 1.52 [0.77, 4.73] | 1.13 [0.67, 2.30] | 9.67 [5.39, 16.71] | <0.001 |
| ALBI | -2.60 [-2.86, -2.15] | -2.67 [-2.91, -2.35] | -1.92 [-2.37, -1.70] | <0.001 |
| APRI | 0.27 [0.18, 0.44] | 0.27 [0.19, 0.46] | 0.29 [0.18, 0.39] | 0.744 |
| NPAR | 1.74 [1.51, 2.13] | 1.61 [1.43, 1.88] | 2.26 [2.05, 2.51] | <0.001 |

Categorical variables are presented as n (%) and compared using Chi-square test or Fisher exact test. Continuous variables are presented as median [interquartile range] and compared using Mann–Whitney U test. P< 0.05, which means they are statistically significant.
