# Supplementary material for: Development and external validation of a machine learning model for predicting gangrenous cholecystitis
Source: Front Med (Lausanne). 2026 Sep 14;13:1930347. doi: 10.3389/fmed.2026.1930347 (PMC13616794; doi:10.3389/fmed.2026.1930347)
Supplement: Supplementary file 3 [file Table3.docx]

**Supplementary Table 3. Baseline Characteristics Comparison between Training and Internal Validation Cohorts.**

| **Variables** | **Training set (N=282)** | **Validation set (N=120)** | **p** |
| --- | --- | --- | --- |
| Gender[n(%)] |  |  | 0.954 |
| Female | 160 (56.74) | 67 (55.83) |  |
| Male | 122 (43.26) | 53 (44.17) |  |
| N.V [n(%)] |  |  | 0.869 |
| No | 171 (60.64) | 71 (59.17) |  |
| Yes | 111 (39.36) | 49 (40.83) |  |
| DM [n(%)] |  |  | 0.055 |
| No | 248 (87.94) | 96 (80.00) |  |
| Yes | 34 (12.06) | 24 (20.00) |  |
| HTN [n(%)] |  |  | 1.000 |
| No | 201 (71.28) | 86 (71.67) |  |
| Yes | 80 (28.37) | 34 (28.33) |  |
| CHD [n(%)] |  |  | 0.640 |
| No | 266 (94.33) | 111 (92.50) |  |
| Yes | 16 (5.67) | 9 (7.50) |  |
| CBDS [n(%)] |  |  | 0.975 |
| No | 230 (81.56) | 97 (80.83) |  |
| Yes | 52 (18.44) | 23 (19.17) |  |
| TP [n(%)] |  |  | 0.087 |
| No | 34 (12.06) | 23 (19.17) |  |
| Yes | 248 (87.94) | 97 (80.83) |  |
| Age (years) | 62.00 [49.25, 71.00] | 64.50 [50.00, 71.00] | 0.967 |
| BMI(kg/m^2^) | 24.80 [22.50, 26.38] | 24.75 [22.00, 27.07] | 0.693 |
| WBC(10^9^/L) | 7.39 [5.48, 11.09] | 7.96 [5.74, 12.35] | 0.179 |
| NEU.(%) | 73.30 [61.92, 86.17] | 72.80 [66.75, 85.72] | 0.332 |
| NEUT (10^9^/L) | 5.30 [3.48, 9.27] | 5.94 [3.70, 10.29] | 0.227 |
| LYM (10^9^/L) | 1.31 [0.88, 1.81] | 1.31 [0.90, 1.77] | 0.964 |
| MONO (10^9^/L) | 0.36 [0.26, 0.56] | 0.38 [0.28, 0.59] | 0.230 |
| Hb (g/L) | 137.00 [126.00, 149.00] | 138.50 [130.00, 147.00] | 0.342 |
| PLT(10^9^/L) | 227.50 [188.00, 270.00] | 233.50 [181.75, 284.25] | 0.320 |
| ALT(U/L) | 26.00 [16.00, 78.25] | 24.00 [14.75, 57.00] | 0.416 |
| AST (U/L) | 25.50 [20.00, 50.00] | 23.00 [18.00, 37.25] | 0.054 |
| GGT (U/L) | 44.88 [23.11, 190.75] | 39.00 [19.03, 173.25] | 0.250 |
| ALP (U/L) | 102.00 [79.00, 142.00] | 97.00 [80.25, 143.25] | 0.709 |
| TBIL (μmol/L) | 17.23 [12.17, 28.84] | 15.80 [11.93, 23.30] | 0.164 |
| DBIL (μmol/L) | 5.25 [3.54, 11.50] | 4.66 [3.76, 8.80] | 0.325 |
| ALB (g/L) | 42.95 [39.58, 45.30] | 42.67 [39.08, 45.28] | 0.786 |
| PT (s) | 11.90 [11.30, 12.60] | 11.85 [11.20, 12.70] | 0.761 |
| INR | 1.04 [0.98, 1.11] | 1.04 [0.98, 1.12] | 0.646 |
| FIB (g/L) | 3.39 [2.68, 4.96] | 3.45 [2.72, 4.93] | 0.488 |
| GBWT (cm) | 0.03 [0.03, 0.05] | 0.03 [0.03, 0.05] | 0.337 |
| T (℃) | 36.50 [36.30, 36.50] | 36.50 [36.50, 36.60] | 0.016 |
| HR(beats/min) | 80.00 [75.00, 89.00] | 79.00 [74.00, 86.25] | 0.226 |
| NLR | 4.32 [2.06, 10.26] | 3.79 [2.53, 10.37] | 0.448 |
| PLR | 175.50 [126.01, 262.00] | 177.20 [139.57, 252.87] | 0.526 |
| PAR | 5.26 [4.48, 6.47] | 5.47 [4.62, 6.74] | 0.300 |
| PHR | 1.61 [1.35, 1.97] | 1.69 [1.35, 2.06] | 0.522 |
| SII | 900.44 [461.09, 2122.96] | 1008.31 [556.11, 2214.86] | 0.303 |
| SIRI | 1.23 [0.65, 4.98] | 1.52 [0.77, 4.73] | 0.241 |
| ALBI | -2.58 [-2.85, -2.15] | -2.60 [-2.86, -2.15] | 0.592 |
| APRI | 0.29 [0.21, 0.60] | 0.27 [0.18, 0.44] | 0.051 |
| NPAR | 1.72 [1.42, 2.04] | 1.74 [1.51, 2.13] | 0.361 |

Categorical variables are presented as n (%) and compared using Chi-square test or Fisher exact test. Continuous variables are presented as median [interquartile range] and compared using Mann–Whitney U test. P < 0.05, which means they are statistically significant
