# Supplementary material for: Development and external validation of a machine learning model for predicting gangrenous cholecystitis
Source: Front Med (Lausanne). 2026 Sep 14;13:1930347. doi: 10.3389/fmed.2026.1930347 (PMC13616794; doi:10.3389/fmed.2026.1930347)
Supplement: Supplementary file 4 [file Table4.docx]

**Supplementary Table 4. Two-Centre Comparison of Baseline Characteristics**

| **Variables** | **Center 1**  **(First Affiliated Hospital)**  **N=402** | **Center 2**  **(Third Affiliated Hospital)**  **N=90** | **P value** | **SMD** |
| --- | --- | --- | --- | --- |
| Gender[n(%)] |  |  | 0.032 | -0.263 |
| Female | 227 (56.47) | 39 (43.33) |  |  |
| Male | 175 (43.53) | 51 (56.67) |  |  |
| N.V [n(%)] |  |  | 0.281 | -0.139 |
| No | 242 (60.20) | 48 (53.33) |  |  |
| Yes | 160 (39.80) | 42 (46.67) |  |  |
| DM [n(%)] |  |  | 0.016 | -0.281 |
| No | 344 (85.57) | 67 (74.44) |  |  |
| Yes | 58 (14.43) | 23 (25.56) |  |  |
| HTN [n(%)] |  |  | 0.001 | -0.381 |
| No | 287 (71.39) | 48 (53.33) |  |  |
| Yes | 115 (28.61) | 42 (46.67) |  |  |
| CHD [n(%)] |  |  | 0.294 | -0.139 |
| No | 377 (93.78) | 81 (90.00) |  |  |
| Yes | 25 ( 6.22) | 9 (10.00) |  |  |
| CBDS [n(%)] |  |  | 0.182 | -0.167 |
| No | 327 (81.34) | 67 (74.44) |  |  |
| Yes | 75 (18.66) | 23 (25.56) |  |  |
| TP [n(%)] |  |  | 0.550 | -0.092 |
| No | 57 (14.18) | 10 (11.11) |  |  |
| Yes | 345 (85.82) | 80 (88.89) |  |  |
| PATH |  |  | 0.586 | -0.079 |
| No | 317 (78.86) | 66 (73.33) |  |  |
| Yes | 85 (21.14) | 24 (26.67) |  |  |
| Age (years) | 63.00  [50.00, 71.00] | 64.50  [55.00, 71.00] | 0.439 | -0.094 |
| BMI(kg/m^2^) | 24.80  [22.50, 26.60] | 24.78  (3.97) | 0.989 | -0.021 |
| WBC(10^9^/L) | 7.67  [5.49, 11.36] | 11.14  [7.31, 16.58] | <0.001 | -0.635 |
| NEU.(%) | 73.10  [63.30, 86.18] | 83.05  [67.50, 90.65] | 0.001 | -0.353 |
| NEUT (10^9^/L) | 5.51  [3.52, 9.54] | 9.21  [4.93, 14.70] | <0.001 | -0.522 |
| LYM (10^9^/L) | 1.31  [0.88, 1.81] | 1.14  [0.68, 1.63] | 0.083 | 0.138 |
| MONO (10^9^/L) | 0.37  [0.27, 0.56] | 0.50  [0.36, 0.71] | <0.001 | -0.277 |
| Hb (g/L) | 137.84  (16.02) | 141.50  [131.00, 150.75] | 0.245 | -0.034 |
| PLT(10^9^/L) | 231.00  [187.00, 273.75] | 234.00  [179.50, 306.00] | 0.379 | -0.202 |
| ALT(U/L) | 25.00  [16.00, 69.75] | 33.50  [18.00, 142.50] | 0.094 | -0.273 |
| AST (U/L) | 25.00  [19.00, 45.00] | 28.00  [18.25, 75.00] | 0.313 | -0.219 |
| GGT (U/L) | 41.44  [21.09, 176.50] | 67.09  [29.17, 291.66] | 0.011 | -0.238 |
| ALP (U/L) | 100.00  [79.00, 142.00] | 110.50  [85.25, 192.25] | 0.218 | -0.268 |
| TBIL (μmol/L) | 16.82  [12.11, 26.99] | 22.75  [13.02, 38.66] | 0.009 | -0.284 |
| DBIL (μmol/L) | 5.10  [3.62, 10.42] | 6.54  [3.82, 17.68] | 0.053 | -0.213 |
| ALB (g/L) | 42.90  [39.40, 45.30] | 42.00  [37.12, 45.12] | 0.244 | 0.150 |
| PT (s) | 11.90  [11.30, 12.60] | 12.20  [11.53, 12.90] | 0.050 | -0.207 |
| INR | 1.04  [0.98, 1.11] | 1.06  [1.00, 1.13] | 0.124 | -0.089 |
| FIB (g/L) | 3.41  [2.69, 4.97] | 4.17  [2.92, 5.88] | 0.029 | -0.221 |
| GBWT (cm) | 0.30  [0.30, 0.50] | 0.50  [0.30, 0.70] | <0.001 | -0.435 |
| T (℃) | 36.50  [36.30, 36.50] | 36.50  [36.40, 36.60] | 0.268 | -0.231 |
| HR(beats/min) | 80.00  [74.25, 88.00] | 80.00  [75.00, 91.50] | 0.667 | -0.035 |
| NLR | 3.91  [2.19, 10.33] | 7.44  [2.83, 19.29] | <0.001 | -0.404 |
| PLR | 175.50  [128.60, 261.79] | 211.34  [135.78, 316.05] | 0.067 | -0.267 |
| PAR | 5.35  [4.50, 6.52] | 5.54  [4.42, 7.02] | 0.259 | -0.262 |
| PHR | 1.63  [1.35, 2.01] | 1.61  [1.31, 2.23] | 0.692 | -0.266 |
| SII | 939.40  [503.35, 2201.14] | 2017.17  [626.27, 3959.76] | <0.001 | -0.588 |
| SIRI | 1.32  [0.67, 4.98] | 4.09  [1.23, 11.49] | <0.001 | -0.309 |
| ALBI | -2.59  [-2.85, -2.15] | -2.42  [-2.79, -1.83] | 0.020 | -0.305 |
| APRI | 0.28  [0.21, 0.54] | 0.30  [0.20, 0.95] | 0.607 | -0.280 |
| NPAR | 1.72  [1.44, 2.06] | 1.94  (0.50) | 0.003 | -0.342 |

Categorical variables are presented as n (%) and compared using Chi-square test or Fisher exact test. Continuous variables are presented as median [interquartile range] and compared using Mann–Whitney U test. SMD = standardized mean difference (Hedges' g for continuous variables, Cohen's h for categorical variables). P < 0.05, which means they are statistically significant.
