# Supplementary material for: Development and external validation of a machine learning model for predicting gangrenous cholecystitis
Source: Front Med (Lausanne). 2026 Sep 14;13:1930347. doi: 10.3389/fmed.2026.1930347 (PMC13616794; doi:10.3389/fmed.2026.1930347)
Supplement: Supplementary file 5 [file Table5.docx]

Supplementary Table 5 Full training hyperparameters of the optimal ANN model

| ****Item**** | ****Detailed Settings**** |
| --- | --- |
| activation | logistic |
| alpha | 0.0001 |
| batch_size | auto |
| beta_1 | 0.9 |
| beta_2 | 0.999 |
| early_stopping | FALSE |
| epsilon | 0 |
| hidden_layer_sizes | (100,) |
| learning_rate | constant |
| learning_rate_init | 0.001 |
| max_fun | 15000 |
| max_iter | 500 |
| momentum | 0.9 |
| n_iter_no_change | 10 |
| nesterovs_momentum | TRUE |
| power_t | 0.5 |
| random_state | 123 |
| shuffle | TRUE |
| solver | adam |
| tol | 0.0001 |
| validation_fraction | 0.1 |
| verbose | FALSE |
| warm_start | FALSE |
